# SARS‐CoV‐2 infection as a potential triggering factor for urticarial vasculitis during pregnancy: A case report

**DOI:** 10.1002/ccr3.4323

**Published:** 2021-06-24

**Authors:** Mohammad Shahidi Dadras, Azadeh Rakhshan, Reem Diab, Fahimeh Abdollahimajd

**Affiliations:** ^1^ Skin Research Center Shahid Beheshti University of Medical Sciences Tehran Iran; ^2^ Clinical Research Development Unit Shohada‐e Tajrish Hospital Shahid Beheshti University of Medical Sciences Tehran Iran; ^3^ Department of Pathology Shohada‐e Tajrish Hospital School of Medicine Shahid Beheshti University of Medical Sciences Tehran Iran

**Keywords:** COVID‐19, pregnancy, SARS‐CoV‐2, urticaria, urticarial vasculitis, vasculitis

## Abstract

During the COVID‐19 pandemic, physicians must maintain a high index of suspicion for COVID‐19 in cases of urticarial vasculitis or other forms of urticaria. This is particularly important for acute presentations in otherwise asymptomatic individuals and pregnant women, where a prompt approach to the patient can prevent undesirable complications.

## Graphical abstract

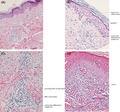

## 1 INTRODUCTION

To the best of our knowledge, there are no published cases of urticarial vasculitis associated with COVID‐19 during pregnancy. Urticarial vasculitis and other forms of urticaria may be alarming signs of COVID‐19, especially in asymptomatic patients presenting acutely with such lesions and among individuals with special conditions like pregnancy.

The coronavirus disease 2019 (COVID‐19) not only manifests as a respiratory illness but also can affect any organ including the skin and vascular system.^1^, ^2^, ^3^ In some patients afflicted with severe COVID‐19, a pattern of microvascular damage has been reported particularly in the lung and skin.^4^ One of the cutaneous manifestations caused by vascular injury is urticarial vasculitis. This condition is characterized by persistent urticarial lesions lasting for more than 24 hours and usually resolving with postinflammatory hyperpigmentation.^5^ Regarding the histopathology, urticarial vasculitis is defined by small vessel vasculitis including perivascular infiltrates of leukocytes, endothelial swelling, red blood cell extravasation, vessel wall necrosis, fibrinoid deposits, and leukocytoclasia.^6^

With this evolving pandemic, there are new and unpredictable skin manifestations in various groups of patients like pregnant women. This issue must be taken seriously to prevent undesirable complications.^1^ Herein, we report the case of a pregnant woman who presented with urticarial vasculitis shortly before the onset of other COVID‐19 symptoms.

## 2 CASE PRESENTATION

**FIGURE 1:**
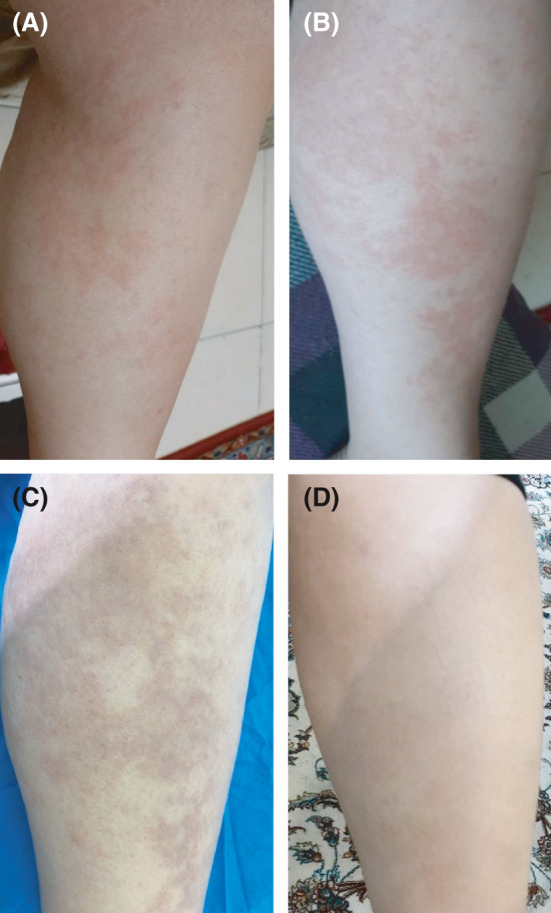
(A, B) Erythematous plaques on both extremities on the second day of disease onset. (C) Erythematous wheals with postinflammatory hyperpigmentation (PIH) on the 7th day. (D) Complete resolution of the skin lesions with mild PIH on the 10th day of treatment

**FIGURE 2:**
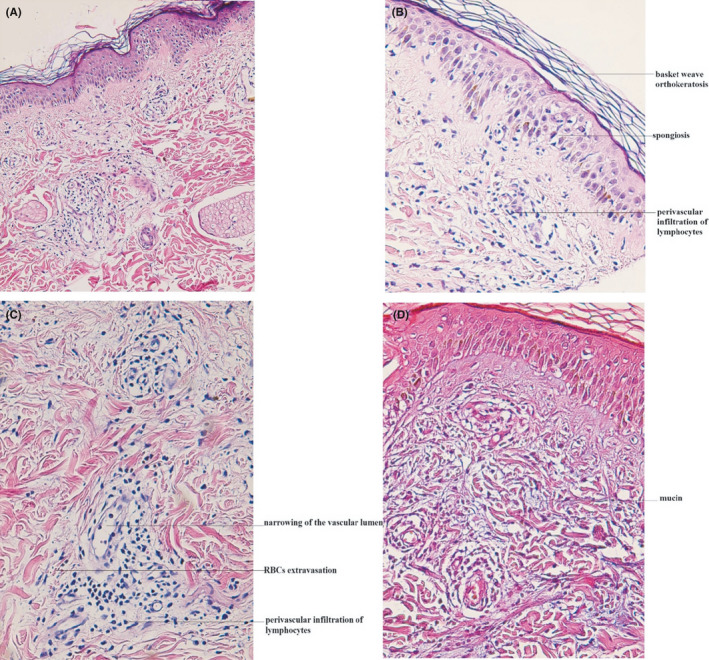
(A‐C) Sections show skin tissue with normal epidermal thickness, mild basket weave orthokeratosis, mild spongiosis, and focal vacuolar interface reaction. There is mild perivascular infiltration of lymphocytes in the upper dermis accompanied by swelling of endothelial cells, narrowing of the vascular lumen, and mild extravasation of red blood cells. A few eosinophils are also present in the infiltrate (H&E, ×200, ×400, and ×400, respectively). (D) Mild interstitial mucin deposition in the upper dermis is noted (Alcian blue stain, ×400). These findings are most in favor of an urticarial vasculopathic reaction associated with viral disease (COVID‐19)

On September 19, 2020, a 30‐year‐old pregnant woman with no specific past medical history presented to our clinic with pruritic, erythematous, indurated wheals on the lower extremities that started to appear one week beforehand (Figure 1). The patient was in the 28th week of pregnancy and was taking iron supplementation and prenatal multivitamins. A skin biopsy was taken with differential diagnoses of urticarial vasculitis, atypical erythema multiforme, and drug reaction. Three days later, the patient started complaining of a sore throat. No fever, myalgia, or other systemic symptoms were reported. Given that her husband was afflicted with COVID‐19, a nasal swab polymerase chain reaction (PCR) test for severe acute respiratory syndrome coronavirus 2 (SARS‐CoV‐2) was requested, which came back positive. Furthermore, the histopathological evaluation of the skin biopsy was compatible with COVID‐19‐associated urticarial vasculitis (Figure 2).

**FIGURE 3:**
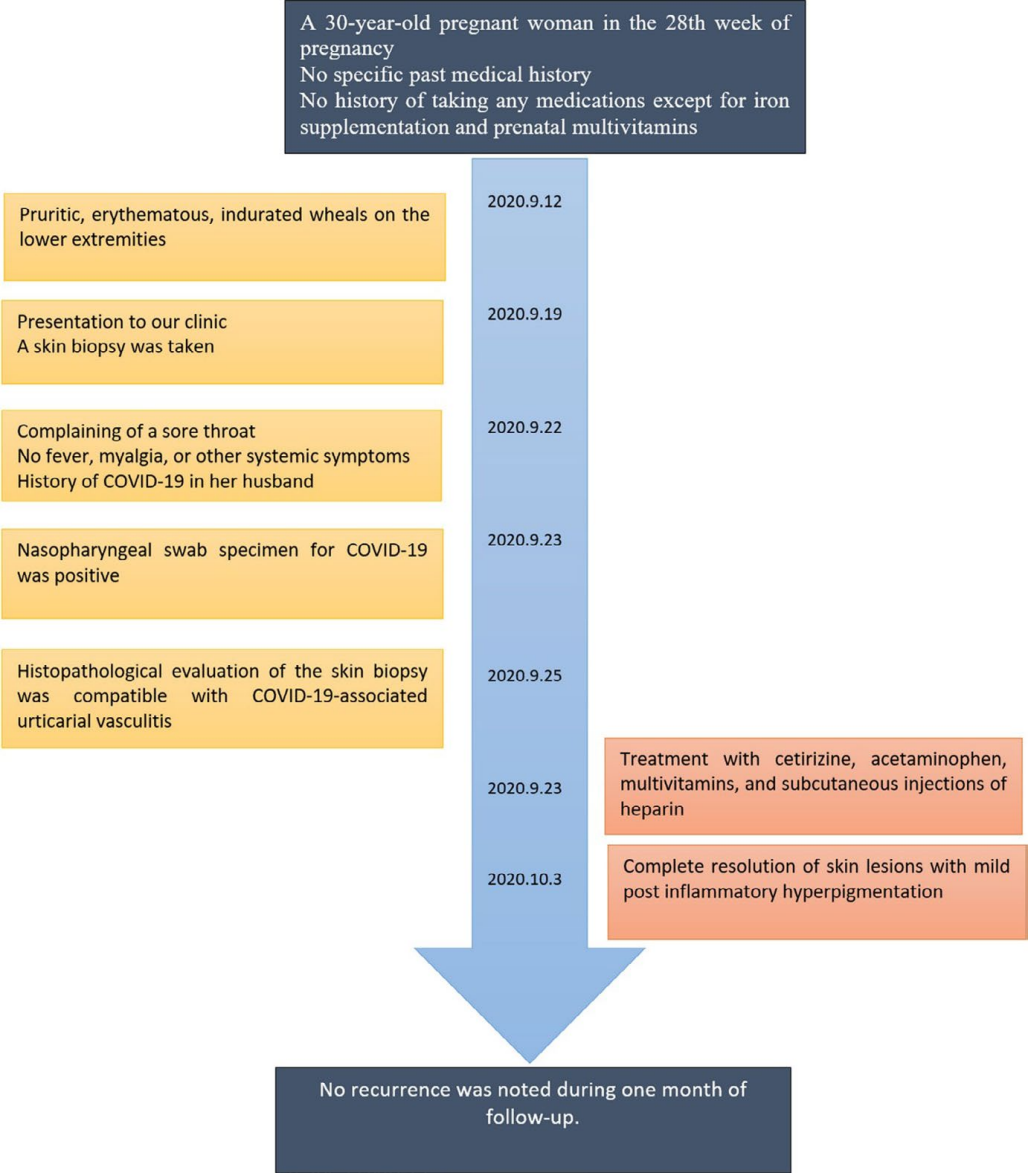
Timeline of clinical presentations, interventions, and outcomes in the patient

The patient was treated with cetirizine, acetaminophen, multivitamins, and subcutaneous injections of heparin. Ten days later, there was complete resolution of skin lesions. No recurrence was noted during one month of follow‐up (Figure 3).

## 3 DISCUSSION

Physicians should make note of the fact that COVID‐19 can present with various clinical manifestations including coagulopathy and vasculopathy, which were not reported in previous coronavirus diseases such as SARS and MERS.^7^ Moreover, the vascular system is one of the main target organs for SARS‐CoV‐2, which has a great tendency to adhere to the membrane of vascular endothelial cells and cause vasculitis, especially when there are predisposing factors such as diabetes mellitus, hypertension, or old age.^1^ Another important factor to be considered is pregnancy, in which significant physiological changes affect the coagulation and cardiovascular systems as well as the immune response to viruses.^8^ Although these changes are important for the immune system to adapt and accept the fetus, the response to infectious agents will also change.^8^

SARS‐CoV‐2 causes endothelial cell injury either directly through cell invasion or indirectly through perivascular inflammation, which promotes endothelial damage.^9^ In addition, the levels of cytokines (particularly interleukin 6) and other phase reactants are increased significantly in SARS‐CoV‐2 infection, leading to endothelial damage.^10^ Endothelial injury and dysfunction, in turn, lead to potentially fatal complications such as venous thromboembolism and multiple organ involvement, meaning that prophylactic anticoagulation should be initiated early, especially in hospitalized patients who have multiple comorbidities and pregnant women.^9^, ^10^

One of the important types of vasculitis is urticarial vasculitis, which represents a type III hypersensitivity reaction associated mainly with drugs, viral infections, and autoimmune diseases.^11^ Lymphocytic vasculitis, endothelial cell swelling, and extravasation of red blood cells have been reported in the histopathologic examinations of urticarial lesions (especially on the extremities) of several patients with COVID‐19.^1^ One of the important differential diagnoses that should be considered in this case is drug reaction. In drug reactions, there is usually a history of recent drug use, and cessation of the causal drug leads to improvement of clinical symptoms.^12^ In this case, the patient was only using iron supplementation and multivitamins, and no new drug was initiated recently. In addition, the histology of drug reaction includes necrotic keratinocytes, papillary dermal edema, red blood cells’ extravasation, and a perivascular infiltrate that is usually composed of lymphocytes and eosinophils^12^

It is important to note that urticaria and urticarial vasculitis could be warning features for COVID‐19 that may be seen in some asymptomatic patients.^13^ Moreover, most pregnant women with COVID‐19 may only have mild symptoms or even no symptoms.^8^ Hence, during this pandemic, it is recommended to request the nasal swab PCR test for SARS‐CoV‐2 in patients newly presenting with urticarial lesions (especially urticarial vasculitis) without any known trigger factors. Moreover, an appropriate and quick diagnosis of these dermatologic findings is significantly important as they can point to an underlying SARS‐CoV‐2 infection. This may result in protective measures to reduce disease transmission and/or initiate appropriate treatments to minimize the risk of thrombus formation in patients suspected of coagulation abnormalities.^13^

Considering the lack of studies on the safety of COVID‐19 vaccination in pregnant women, the alternative option of using hydroxychloroquine as a prophylactic medication to prevent severe complications and gestational problems in this population has been discussed.^14^ Many studies have confirmed the antiviral and anti‐inflammatory features of hydroxychloroquine, which may be useful in preventing SARS‐CoV‐2 infection in pregnant women, thereby averting undesirable complications and risks for the mother and the fetus.^14^

To the best of our knowledge, this report is the first case of urticarial vasculitis in a pregnant woman with COVID‐19. An additional novelty of this case is the identification of mucin deposition in the upper dermis, which may be a new histological feature of vasculitis seen in COVID‐19. We believe that similar cases are being overlooked due to a lack of performing skin biopsies in patients with COVID‐19. Hence, we recommend that skin biopsies and further evaluation are considered for suspicious cases. Also, we recommend further clinical trials on the use of hydroxychloroquine as a preventive tool to stop COVID‐19 spread among pregnant women considering its antiviral and anti‐inflammatory properties.

## 4 CONSENT TO PARTICIPATE AND FOR PUBLICATION

Written informed consent was obtained from the patient for publication of this case report and any accompanying images.

## CONFLICT OF INTEREST

The authors declare no conflict of interest.

## AUTHOR CONTRIBUTIONS

MSD and FA: were involved in the diagnosis and management of the patient and have been responsible for the clinical part of the manuscript. AR: reported the result of histopathological evaluation. RD: did literature review and drafted the manuscript. RD, FA and MSD: were responsible for final editing of the manuscript. All authors have read and approved the final manuscript.

## Data Availability

All data are included in this published report.
